# THE HUMAN-ANIMAL BOND: SOCIAL NETWORK COMPOSITIONS, ANIMAL COMPANIONS, AND HEALTH

**DOI:** 10.1093/geroni/igz038.726

**Published:** 2019-11-08

**Authors:** Raeann G LeBlanc

**Affiliations:** University of Massachusetts Amherst, Amherst, Massachusetts, United States

## Abstract

Animal companionship has been found to be positively related to health, though less is known about the features of social networks that include animal companions and how these relate to health outcomes. The purpose of this study was to explore the relationship between social network composition including animal companionship and health. A mixed methods cross-sectional descriptive, correlation study design was used. Eighty-nine people age sixty-five and older, living in the community, managing multiple chronic conditions, participated in telephone interviews. Animal companionship was common (42.7%) among the sample (66% lived alone) with at least one animal companion (M=1.57, SD=.903) and associated with improved health function (IADL scores) (r=.234, p=.028). Animal companionship correlated positively with health (SF12 General Health Scores) (r=.210, p=.048). Animal companionship is an important feature in social networks of older people that influences health. Social supports maintain these relationships and the animal human bond.

